# Verifying Physiological and Biomechanical Parameters during Continuous Swimming at Speed Corresponding to Lactate Threshold

**DOI:** 10.3390/sports8070095

**Published:** 2020-06-30

**Authors:** Gavriil G. Arsoniadis, Ioannis S. Nikitakis, Petros G. Botonis, Ioannis Malliaros, Argyris G. Toubekis

**Affiliations:** 1 Division of Aquatic Sports, School of Physical Education and Sports Science, National and Kapodistrian University of Athens, Dafne, 17237 Athens, Greece; garsoniadis@phed.uoa.gr (G.G.A.); inikitak@phed.uoa.gr (I.S.N.); pboton@phed.uoa.gr (P.G.B.); gmalliaros@phed.uoa.gr (I.M.); 2 Sports Performance Laboratory, School of Physical Education and Sport Science, National and Kapodistrian University of Athens, Dafne, 17237 Athens, Greece

**Keywords:** lactate threshold, continuous swimming, physiological responses, biomechanical parameters, validity

## Abstract

The purpose of this study was to verify the physiological responses and biomechanical parameters measured during 30 min of continuous swimming (T30) at intensity corresponding to lactate threshold previously calculated by an intermittent progressively increasing speed test (7 × 200 m). Fourteen competitive swimmers (18.0 (2.5) years, 67.5 (8.8) kg, 174.5 (7.7) cm) performed a 7 × 200 m front crawl test. Blood lactate concentration (BL) and oxygen uptake (VO_2_) were determined after each 200 m repetition, while heart rate (HR), arm-stroke rate (SR), and arm-stroke length (SL) were measured during each 200 m repetition. Using the speed vs. lactate concentration curve, the speed at lactate threshold (sLT) and parameters corresponding to sLT were calculated (BL-sLT, VO_2_-sLT, HR-sLT, SR-sLT, and SL-sLT). In the following day, a T30 corresponding to sLT was performed and BL-T30, VO_2_-T30, HR-T30, SR-T30, and SL-T30 were measured after the 10th and 30th minute, and average values were used for comparison. VO_2_-sLT was no different compared to VO_2-_T30 (*p* > 0.05). BL-T30, HR-T30, and SR-T30 were higher, while SL-T30 was lower compared to BL-sLT, HR-sLT, SR-sLT, and SL-sLT (*p* < 0.05). Continuous swimming at speed corresponding to lactate threshold may not show the same physiological and biomechanical responses as those calculated by a progressively increasing speed test of 7 × 200 m.

## 1. Introduction

Progressive discontinuous swim protocols, such as a 7 × 200 m progressively increasing speed test, are commonly used to evaluate both physiological [1,2] and biomechanical [3] characteristics in swimming. More specifically, a 7 × 200 m test is used to identify aerobic training intensity domains and subsequent changes during a year-round training plan [4]. The identification of training intensity domains requires drawing a speed vs. blood lactate concentration curve and calculating specific aerobic indices, such as speed corresponding to first and second lactate thresholds [5,6]. The sLT (speed at lactate threshold) is one of the most frequently used indices to assess swimming endurance capacity [1,7], and several methods are utilized for its calculation [8].

In swimming, the most frequently used method for sLT calculation is x-axis projection of the intersection of two lines connecting the three higher and four lower points of the speed lactate curve [7]. Subsequently, biomechanical or physiological parameters corresponding to sLT may be calculated to provide additional information for coaches (i.e., arm-stroke rate (SR), arm-stroke length (SL), heart rate (HR), and blood lactate concentration (BL) corresponding to sLT). However, all suggested methods used for sLT calculation present errors in estimation, and this may be transferred to the training pace prescription of swimmers [9]. The validity of sLT is tested by calculating the speed corresponding to the maximum lactate steady state (MLSS: maximum lactate concentration that can be maintained constant during continuous exercise; [10,11]), which is a time-consuming test for verification and implies that sLT may be used during continuous swimming training. Whatever the case, there is a need to verify the calculated sLT and corresponding physiological and biomechanical variables obtained after a 7 × 200 m test during a continuous swimming training set so as to increase precision in the control of the training load and improve swimming performance [7,12]. A 30 min duration is an appropriate and acceptable time limit to compare variations in biomechanical and physiological parameters in continuous swimming [7]. Using a prescribed sLT speed, a specific response in physiological (HR, BL) or biomechanical variables (SR, SL) is expected, as these parameters are interconnected [13]. Therefore, verifying this information is important before coaches plan a training set. However, to our knowledge, there has been no previous study to verify the calculated sLT using a continuous 30 min of swimming.

Thus, the purpose of the current study was to verify the physiological responses and biomechanical parameters during continuous swimming at intensity corresponding to the lactate threshold previously calculated by an intermittent progressively increasing speed test (7 × 200 m). We hypothesized that the calculated parameters would be verified during the continuous swimming effort.

## 2. Materials and Methods

### 2.1. Participants

Fourteen regional and national level male and female swimmers (ten sprinters and four middle-distance swimmers) that specialized in competitive distances of 100, 200, and 400 m volunteered to participate in the study (Table 1). Participants had a training background of 9.9 (1.8) years and they participated in daily training (6 days per week) with a duration of approximately two hours per session. Participants were randomly selected from local swimming clubs after getting agreement from parents and coaches. Swimmers were not consuming nutritional supplements during the testing period; they were asked to consume the same diet two days before the trials. Participants were instructed to avoid alcohol or caffeine consumption two days before each testing session. Each participant and his or her legal guardian provided written informed consent after receiving thorough explanation of the study. The local institutional review board approved the experimental procedures (approval no: 1029/6/12/2017) in accordance with Helsinki declaration for human subjects.

### 2.2. Study Design

**Figure 1:**
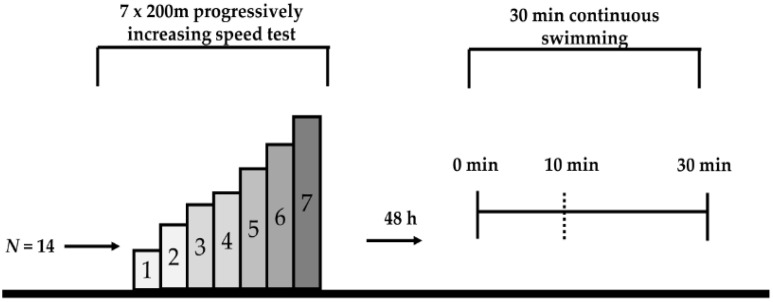
Experimental design of the current study; 7 × 200 m: 7 repetitions of 200 m front crawl, h: hours.

Physiological and biomechanical parameters calculated during a progressively increasing speed swimming test (mean overall time: ~45 min) were compared to those measured during 30 min of continuous swimming. Swimmers were tested in two sessions 48 h apart (Figure 1), and all tests were completed at the same time of the day for each swimmer (between 14:00 to 16:00). Prior to the first testing session, body mass and height were measured (Seca, Hamburg, Germany). One day prior to the first testing session, as well as during the two days separating the two testing sessions, swimmers participated in an easy (BL: ~2 mmol L^−1^) low volume endurance training (~3000 to 4000 m). The study was conducted during the specific preparation mesocycle of training. All swimming tests were performed using front crawl in a 25 m indoor swimming pool with a constant water temperature of 25 °C to 26 °C and 60% ambient humidity.

### 2.3. Progressively Increasing Swimming Speed Test and Parameters Calculation

All swimmers participated in a standardized swimming warm-up, and 10 min later performed a 7 × 200 m front crawl test at intensities calculated using the most recent 200 m race time and corresponding to 60%, 70%, 75%, 80%, 85%, 90%, and maximum effort. Participants were familiarized with the pace of the first two repetitions during a previous training session. During testing, one of the experimenters walked alongside the swimming pool providing guidance during each 200 m repetition. During the 7 × 200 m test, each repetition started every five minutes and 30 s with a push-off start from within the water [1]. Fingertip blood samples were collected after each repetition and were analyzed for BL (Lactate Scout^+^, SensLab GmbH, Leipzig, Germany). Immediately after the completion of each 200 m repetition, a mouthpiece and a nose clip were attached to swimmers during recovery. Expired air collected during the first 20 s of recovery was analyzed for oxygen uptake (VO_2_) using a portable gas analyzer (VO2000, Med Graphics, Saint Paul, MN, USA.; [14]). HR was recorded continuously using telemetry (s610i; Polar Electro, Kempele, Finland). A vest was used to keep the transmitter attached to each swimmer’s chest while swimming. sLT and the respective BL-sLT were calculated by the x-axis projection of the intersection of lines connecting the three higher and four lower points of the speed lactate curve (BL-sLT: mean *R*^2^ = 0.97 (0.02), mean *r* = 0.99 (0.01); 1). VO_2-_sLT and HR-sLT were calculated by interpolation using linear regression of swimming speed versus VO_2_ (mean *R*^2^ = 0.99 (0.02), mean *r* = 0.99 (0.01)) or HR (mean *R*^2^ = 0.99 (0.01), mean *r* = 0.99 (0.01). During the 7 × 200 m test, SR was calculated by the time (T) used to complete three arm-stroke cycles (180·T^−1^) and SL was calculated by dividing swimming speed every 50 m (V) by SR. The time for three arm-stroke cycles was recorded using a handheld chronometer. SR-sLT and SL-sLT were calculated by interpolation using the best fit regression line of SR and SL versus swimming speed during the 7 × 200 m test (SR: mean *R*^2^ = 0.98 (0.02), mean *r* = 0.99 (0.01); SL: mean *R*^2^ = 0.96 (0.04), mean *r* = 0.98 (0.02)).

### 2.4. Continuous Swimming Session with Constant Speed

The swimmers participated in a continuous swimming session 48 h after the completion of the 7 × 200 m test. Continuous swimming speed session was completed after a standardized warm-up that consisted of 1000 m of swimming (400 m slow swimming at 60% intensity, 4 × 50 m front crawl kicks, 4 × 50 m front crawl drills, and 4 × 50 m front crawl swim with progressively increasing speed). Ten minutes after warm-up, the swimmers started a T30 at a constant speed corresponding to sLT. During T30, the swimmers kept the individual sLT speed constant while guided by a sound signal emitted by a transmitter placed next to the ear and under the swimming cap (FINIS tempo pro, Finis Inc., Livermore, CA, USA). The swimmers were instructed to adjust their speed in order to touch the wall at each sound signal. Additionally, one of the experimenters recorded the time for each 50 m split (HS-80; CASIO, Guangzhou, China). BL-T30 and VO_2_-T30 were measured after the 10th and 30th minute of T30, while HR-T30 was recorded continuously. SR-T30 and SL-T30 were measured every 50 m during the T30 continuous swimming session. The 10th min was used to identify variations in physiological adjustments compared to the 30th min. Nevertheless, the mean values of BL, HR, SR, SL, and speed (s-T30) measured during the T30 were used for the statistical analysis. 

### 2.5. Statistical Analysis

The student *t*-test for dependent samples was used to examine the differences in physiological and biomechanical parameters calculated during the 7 × 200 m progressively increasing speed test and those measured in T30. Specifically, comparisons between sLT, BL-sLT, VO_2_-sLT, HR-sLT, SR-sLT, SL-sLT vs. s-T30, BL-T30, VO_2_-T30, HR-T30, SR-T30, SL-T30, respectively, were applied. Pearson *r* correlation coefficient was used to examine the relationship between the calculated values from a 7 × 200 m test with that measured during T30. The effect size for paired comparisons using the pooled standard deviation as denominator was calculated with Cohen’s *d* [15]. The effect size was considered trivial if the absolute value of Cohen’s *d* was less than 0.20, small if it was between 0.20 and 0.50, medium if it was between 0.50 and 0.80, and large if it was greater than 0.80. The 95% confidence limits (95% CL) were also calculated for the mean differences between parameters. G-Power 3.1.9.4 software [16] was used to examine the power of analysis. Considering the sample size in the current study (*N* = 14), an *ES* of 0.80 was required to get a statistical power value greater than 0.80. For the estimation of agreement between parameters, Bland and Altman plots were used [17]. SPSS software (v.23, SPSS Inc., Chicago, IL, USA) was used for data analysis. Data are presented as mean and standard deviation (SD). Statistical significance was set at *p* < 0.05.

## 3. Results

### 3.1. Swimming Speed between Tests

sLT calculated after the 7 × 200 m test was similar with s-T30 (sLT: 1.317 (0.078) vs. s-T30: 1.316 (0.082) m·s^−1^, mean difference (*SD*): −0.002 (0.010) m·s^−1^, 95% CL: −0.008, 0.005·m·s^−1^, *d =* −0.02, *p =* 0.634). A significant correlation was observed between sLT and s-T30 (*R*^2^ = 0.97, *r* = 0.98, *p* = 0.001).

### 3.2. Comparison of Physiological Variables between Tests

**Figure 2:**
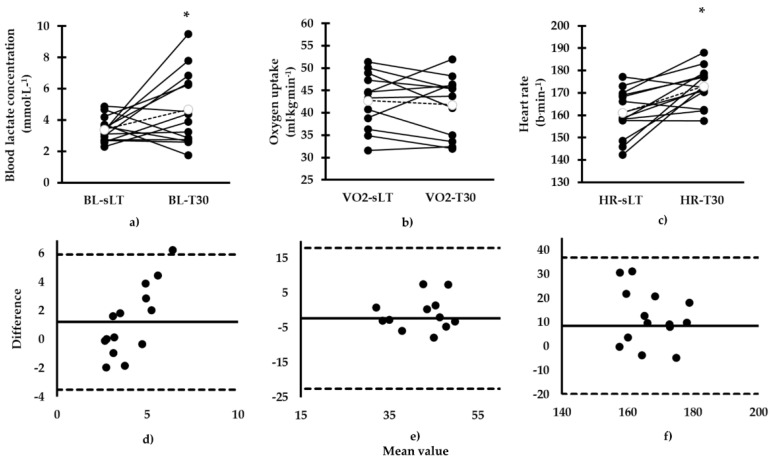
Comparison between calculated and measured parameters in the two tests, (**a**) blood lactate concentration (BL), (**b**) oxygen uptake (VO_2_) (*N* = 12), (**c**) heart rate (HR). Individual values corresponding to BL-sLT, VO_2_-sLT, and HR-sLT are compared with BL-T30, VO_2_-T30, and HR-T30, respectively. Bland and Altman plots of mean vs. difference of two tests is presented in (**d**) BL-sLT and BL-T30, (**e**) VO_2_-sLT and VO_2_-T30, (*N* = 12), and (**f**) HR-sLT and HR-T30. Units of measure in (**d**), (**e**), and (**f**) are not shown for clarity and are the same as in the corresponding figure (**a**), (**b**), and (**c**) panels. * *p* < 0.05 between BL-sLT and BL-T30, VO_2_-sLT and VO_2_-T30, and between HR-sLT and HR-T30. sLT: corresponding to speed at lactate threshold; T30: 30 min of continuous swimming.

Measured BL-T30 was higher compared to BL-sLT (BL-T30: 4.7 (2.3) vs. BL-sLT: 3.4 (0.8) mmol·L^−1^, mean difference (*SD*): 1.3 (2.4) mmol·L^−1^, 95% CL: 0.00, 2.35 mmol·L^−1^, *d* = 0.83, *p* = 0.05; Figure 2), and these variables were not correlated (*R*^2^ = 0.001, *r* = 0.03, *p =* 0.92). A Bland and Altman plot indicated agreement between calculated and measured values (Figure 2). During continuous swimming, HR-T30 was higher compared to HR-sLT (HR-T30: 173 (8) vs. HR-sLT: 161 (10) b·min^−1,^ mean difference (*SD*): 11 (11) b·min^−1^, 95% CL: 6, 18 b·min^−1^, *d* = 1.24, *p* = 0.02; Figure 2), and these variables were not correlated (*R*^2^ = 0.06, *r* = 0.25, *p* = 0.39). Agreement between calculated HR-sLT and measured HR-T30 values was observed (Figure 2). Moreover, VO_2_-T30 was not different compared to VO_2_-sLT (VO_2_-T30: 41.7 (6.8) vs. VO_2_-sLT: 42.8 (6.2) ml·kg^−1^·min^−1^, mean difference (*SD*): −0.88 (4.5) ml·kg^−1^·min^−1^, 95% CL: −3.22, 1.45 ml·kg^−1^·min^−1^, *d* = −0.16, *p* = 0.43) and these parameters were correlated (*R*^2^ = 0.53, *r* = 0.73, *p* = 0.00). A Bland and Altman plot showed agreement between calculated VO_2_-sLT and measured VO_2_-T30 values (Figure 2).

### 3.3. Comparison of Biomechanical Variables between Tests

**Figure 3:**
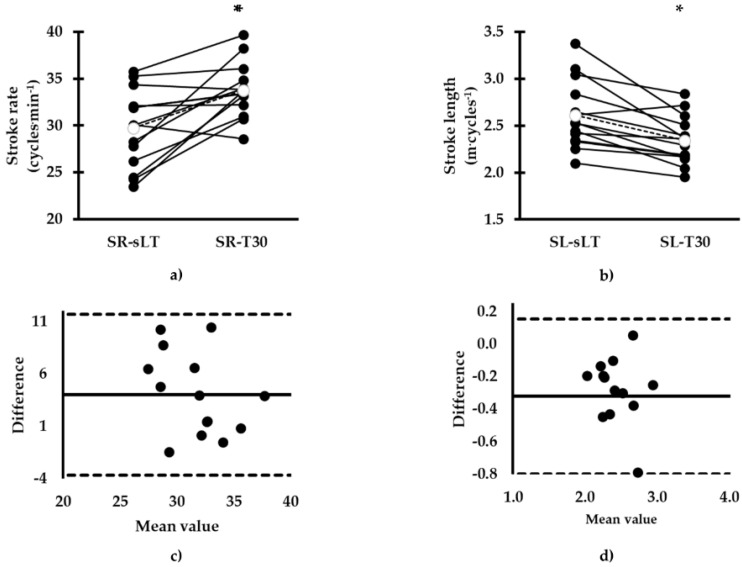
Comparison between calculated and measured parameters in the two tests, (**a**) comparison of arm-stroke rate (SR), (**b**) comparison of arm-stroke length (SL). Individual values corresponding to SR-sLT and SL-sLT are compared with SR-T30 and SL-T30, respectively. Bland and Altman plots of mean vs. difference of the two tests is presented in (**c**) SR-sLT vs. SR-T30, (**d**) SL-sLT vs. SL-T30. Units of measure in (**c**) and (**d**) are not shown for clarity and are the same as in the corresponding (**a**) and (**b**) panel. * *p* < 0.05 between SR-sLT and SR-T30 and between SL-sLT and SL-T30. sLT: corresponding to speed at lactate threshold; T30: 30 min of continuous swimming.

During T30, the measured SR-T30 was higher compared to SR-sLT (SR-T30: 33.8 (2.9) vs. SR-sLT: 29.7 (4.1) cycles·min^−1^, mean difference (*SD*): 4.0 (3.9) cycles·min^−1^, 95% CL: 1.97, 6.07 cycles·min^−1^, *d* = 1.15, *p* = 0.002; Figure 3). SR-T30 and SR-sLT were not correlated (*R*^2^ = 0.16, *r* = 0.41, *p* = 0.15). A Bland and Altman plot indicated agreement between calculated SR-sLT and measured SR-T30 values (Figure 3). Measured SL-T30 was lower compared to SL-sLT (SL-T30: 2.3 (0.3) vs. SL-sLT: 2.6 (0.4) m·cycles^−1^, mean difference (SD): −0.3 (0.2) m·cycles^−1^, 95% CL: −0.40, −0.14 m·cycles^−1^, *d* = −0.88, *p* = 0.001; Figure 3) and these parameters were correlated (*R*^2^ = 0.55, *r* = 0.74, *p* = 0.003). A Bland and Altman plot showed agreement between calculated SL-sLT and measured SL-T30 values (Figure 3).

## 4. Discussion

The purpose of the current study was to verify the physiological and biomechanical parameters measured during continuous constant speed swimming corresponding to lactate threshold with those calculated after a 7 × 200 m test. The calculated sLT was successfully maintained during a T30 session. Calculated VO_2_ was similar to measured VO_2_, while a higher BL, HR, and SR and lower SL were recorded during T30. Bland and Altman plots indicated agreement, although a great bias was observed for all the physiological and biomechanical parameters.

A similar speed compared to sLT was expected in T30 on account of our experimental design. This is because swimmers were guided to follow a constant speed using audio signals. While maintaining the required speed during T30, some physiological adjustments were made by the swimmers. Increased BL and HR were observed during T30, and several factors may have contributed to this increment. First, we should examine the validity of sLT calculation with the method used in the present study. Previous studies report that using the intersection of two lines provides a speed corresponding to lactate threshold similar to MLSS [7]. However, the number of repetitions, the speed increment, and the duration of each repetition may influence the calculation of sLT [7]. Indeed, several methods, mathematical models, and various discontinuous protocols may indicate a different lactate threshold, which is not always similar to MLSS [9,18,19]. In fact, a methodological error of 2.0–2.5% in MLSS calculation should be considered [20]. This is because speed increments of this range are normally used when sLT is compared to MLSS [21]. In this case, a 2.0–2.5% lower speed compared to sLT may induce lower BL in T30, similar to sLT lactate values in the present study. Considering the above, a 2.5% error in calculating sLT should be expected even in studies reporting a valid estimation.

A second factor that needs to be mentioned is that lactate threshold as well as MLSS may present lactate concentration at a range of 2 to 8 mmol∙L^−1^ [22]. In such a case, the swimmers in the current study showed increased lactate values in sLT but it is likely that they were still below or at their MLSS. Despite the limitation that MLSS was not measured, to allow a better understanding of BL-sLT and BL-T30 differences in the present study, it is expected that metabolic/physiological characteristics determine an athlete’s ability to sustain a long duration effort. Endurance athletes are more efficient at maintaining long duration efforts with lower BL as opposed to sprint-oriented athletes at comparable relative exercise intensity [23]. Supporting this, Skorski et al. [24] found a 6.3% to 7.3% higher BL response in short-distance competitive swimmers during training sets such as 5 × 400 m or 5 × 200 m with constant speed corresponding to lactate threshold, suggesting that sLT may induce variable BL response during endurance training sets. In the present study, participants were mainly sprint-oriented and showed 27.3% higher BL than expected during continuous swimming in T30. In this case, the calculated sLT was probably not representing their steady physiological conditions leading to increased lactate production. Moreover, swimmers may show difficulty in maintaining a constant speed for more than 20 min, especially if they are not accustomed to do so, but may be able to maintain the same speed for longer durations in interval training set [25]. Nonetheless, a previous study also found a slightly higher BL during continuous swimming than predicted by 7 × 400 m or 7 × 200 m tests [7]. It seems that continuous effort may correspond to higher exercise intensity, as it has confirmed by higher physiological responses or by the inability to sustain constant speed for a long period [25].

A higher HR was observed during T30, indicating a higher effort during continuous swimming. A higher HR was measured in continuous exercise compared to that calculated corresponding to lactate threshold after a 7 × 200 m test in a previous study [7]. In the above study, increased swimming distance was accompanied by higher HR despite maintaining similar speed [7]. However, in the current study, some of the swimmers may not have reached a steady HR in all stages of the 7 × 200 m test because of the short time needed to complete 200 m repetitions, especially in the last stages (i.e., 130–160 s), thus underestimating the predicted sLT-HR value. Confirming the above information, Fernandes et al. [7] reported 2% (4 b·min^−1^) higher HR corresponding to lactate threshold using 400 m compared to 200 m stages [7]. However, in the current study, a greater HR difference (11 b·min^−1^) was observed between HR-sLT and HR-T30, possibly attributed to the training status and specialty of the swimmers. Additionally, a likely HR drift towards the last minutes of exercise attributed to cardiovascular adjustments during the long exercise duration in T30 cannot be excluded [26]. In contrast to BL and HR, VO_2_-T30 was no different compared to VO_2_-sLT. It has been indicated that oxygen uptake reaches values between ~80–100% of VO_2peak_ at the end of an endurance training set with a duration of 15 to 30 min [25,27]. Specifically, Pelarigo et al. [27] found constant VO_2_ values that were ~85% of VO_2max_, similar to the current study (85.5%). A combination of steady VO_2_ response and increased BL during a continuous exercise may be observed during continuous efforts or in very heavy exercise intensity domains [28].

SR-T30 was increased whereas SL-T30 was decreased compared to SR-sLT and SL-sLT, respectively. Such changes are associated with an increased energy cost [29]. It is possible that the swimmers managed to adjust the applied force during each arm-stroke by increasing the relative duration of propulsive phases in order to maintain the required speed [30]. Similar results have been reported in a previous study in which less experienced athletes presented a decrease in SL with a concomitant increase in SR, despite swimming at a higher speed (by 2.5%) compared to MLSS [31]. On the contrary, Dekerle et al. [32] reported stability in SL during metabolically steady conditions in well-trained competitive swimmers. However, SL decreased at speeds above lactate threshold [21]. It seems that swimmers in the present study were exercising slightly above steady metabolic conditions, and then they were forced to alter their mechanics to maintain the required speed. These alterations in mechanics aim to overcome hydrodynamic drag and may lead to increments in metabolic response.

The abovementioned differences in physiological and biomechanical parameters indicate that the calculated parameters may not always correspond to the measured values during continuous swimming. However, this is in contrast to the observed agreement between BL-sLT, VO_2_-sLT, HR-sLT, SR-sLT, and SL-sLT and BL-T30, VO_2_-T30, HR-T30, SR-T30, and SL-T30, respectively, as indicated by Bland and Altman plots and has also been confirmed previously in a homogenous group of female middle- and long-distance swimmers [33]. Despite the observed agreements presented in the current study, the range of physiological and biomechanical differences observed is great. In this case, we cannot accept that calculated physiological and biomechanical parameters obtained by the intermittent protocol used in this study can predict corresponding ones during continuous long duration swimming. We should consider that BL-sLT, VO_2_-sLT, and HR-sLT as well as SR-sLT and SL-sLT were calculated by equations obtained by the best fit of these parameters versus swimming speed, thus reducing the error of calculation. However, this was not reflected in the measured values, indicating that the observed differences represent real physiological and biomechanical gaps between predicted and measured variables. Further research may examine various mathematical models for lactate threshold calculation in swimmers.

## 5. Conclusions

The physiological and biomechanical parameters calculated by a progressively increasing swimming speed test corresponding to sLT may not be verified during continuous 30 min swimming in sprint and middle-distance swimmers. Swimmers maintaining constant speed corresponding to the second lactate threshold in a long duration 30 min swimming present individualized biomechanical and physiological adjustments that may not reflect the expected responses. In this case, an additional test for verification or a different mathematical model of lactate threshold calculation may be required to provide a valid guidance of training pace. Coaches should be aware that the individual data obtained by a progressively increasing speed test should be examined thoroughly and tested in training practice before planning a training set. 

## Figures and Tables

**Table 1:** Anthropometrics and performance characteristics of competitive swimmers. The data are presented as mean values and standard deviation (*SD*) for both males and females and for each gender separately.

Variables	*N* = 14 Swimmers (Male and Female)	*N* = 10 Male Swimmers	*N* = 4 Female Swimmers
Age (years)	18.0 (2.5)	17.5 (2.4)	19.2 (2.6)
Body mass (kg)	67.5 (8.8)	69.7 (8.1)	60.3 (8.7)
Height (cm)	174.5 (7.7)	175.6 (7.3)	171.9 (9.3)
Time (s) 200 m front crawl	131.6 (7.3)	129.2 (7.3)	137.6 (2.5)
FINA points 200 m front crawl	513.6 (66.0)	503.3 (75.0)	539.5 (28.2)
FINA points (best style)	564.0 (109.3)	540.4 (119.4)	623.0 (49.5)
Competitive experience (years)	9.9 (1.8)	9.5 (1.9)	10.8 (1.5)

FINA: Fédération Internationale de NatationAmateur.

## List of Abbreviations

7 × 200 m:	Progressively increasing speed test
T30:	30 min of continuous swimming
sLT:	Speed corresponding to the second lactate threshold
BL:	Blood lactate concentration
VO_2_:	Oxygen uptake
HR:	Heart rate
SR:	Arm-stroke rate
SL:	Arm-stroke length
BL-sLT:	Blood lactate concentration corresponding to the second lactate threshold
VO_2_-sLT:	Oxygen uptake corresponding to the second lactate threshold
HR-sLT:	Heart rate corresponding to the second lactate threshold
SR-sLT:	Arm-stroke rate corresponding to the second lactate threshold
SL-sLT:	Arm-stroke length corresponding to the second lactate threshold
BL-T30:	Blood lactate concentration during 30 min of continuous swimming
VO_2_-T30:	Oxygen uptake during 30 min of continuous swimming
HR-T30:	Heart rate during 30 min of continuous swimming
SR-T30:	Arm-stroke rate during 30 min of continuous swimming
SL-T30:	Arm-stroke length during 30 min of continuous swimming
